# Correction: Validation and applicability of the music ear test on a large Chinese sample

**DOI:** 10.1371/journal.pone.0300208

**Published:** 2024-03-04

**Authors:** Xiaoyu Wang, Xiubo Ren, Shidan Wang, Dan Yang, Shilin Liu, Meihui Li, Mingyi Yang, Yintong Liu, Qiujian Xu

The order of the affiliations is incorrect. Please view the correct affiliation order below:

Xiaoyu Wang^2^, Xiubo Ren^2^, Shidan Wang ^2^, Dan Yang^2^, Shilin Liu^2^, Meihui Li^2^, Mingyi Yang^2^, Yintong Liu^2^, Qiujian Xu^1,2*^

**1** School of Arts and Design, Yanshan University, 066000, Qinhuangdao, China, **2** Music College, Catholic University of Daegu, 13–13 Hayang-ro, Hayang-eup, Gyeongsan-si, Gyeongsangbuk-do, Republic of Korea.
