# Supplementary material for: Validation and applicability of the music ear test on a large Chinese sample
Source: PLoS One. 2024 Feb 7;19(2):e0297073. doi: 10.1371/journal.pone.0297073 (PMC10849222; doi:10.1371/journal.pone.0297073)
Supplement: S1 File — (DOCX) [file pone.0297073.s001.docx]

**Timeline**

3, 2023 Conducting a literature review to identify research topic

4, 2023 Conducting translations of tests

5-6, 2023 Conducting pre-test experiments and modifying the test content

7-11, 2023 Completing and submitting the registered report

11-12, 2023 Refinement of pre-experimental results and revision of the registration report manuscript based on the review comments

1, 2024 (If there is no need for revision) conducting the tests among the sample

2, 2024 Analyzing the test data to compose the results section

3, 2024 Completing the drafting of the discussion section and developing the first draft

4, 2024 Modifying and submitting the final manuscript
